# Multi-objective reward generalization: improving performance of Deep Reinforcement Learning for applications in single-asset trading

**DOI:** 10.1007/s00521-023-09033-7

**Published:** 2023-10-05

**Authors:** Federico Cornalba, Constantin Disselkamp, Davide Scassola, Christopher Helf

**Affiliations:** 1 Institute of Science and Technology Austria (ISTA), Am Campus 1, 3400 Klosterneuburg, Austria; 2 Department of Mathematical Sciences, University of Bath, Bath, UK; 3 Trality GmbH, Kirchengasse 18, 1070 Vienna, Austria; 4 Department of Mathematics and Geosciences, University of Trieste, Trieste, Italy

**Keywords:** Deep Reinforcement Learning, Multi-objective generalization, Multi-task learning, Stock trading, Cryptocurrency trading, Discount factor generalization

## Abstract

We investigate the potential of Multi-Objective, Deep Reinforcement Learning for stock and cryptocurrency single-asset trading: in particular, we consider a Multi-Objective algorithm which generalizes the reward functions and discount factor (i.e., these components are not specified a priori, but incorporated in the learning process). Firstly, using several important assets (BTCUSD, ETHUSDT, XRPUSDT, AAPL, SPY, NIFTY50), we verify the reward generalization property of the proposed Multi-Objective algorithm, and provide preliminary statistical evidence showing increased predictive stability over the corresponding Single-Objective strategy. Secondly, we show that the Multi-Objective algorithm has a clear edge over the corresponding Single-Objective strategy when the reward mechanism is sparse (i.e., when non-null feedback is infrequent over time). Finally, we discuss the generalization properties with respect to the discount factor. The entirety of our code is provided in open-source format.

## Introduction

Deep Reinforcement Learning (RL) is a subfield of machine learning specifically designed to handle learning processes for problems which involve a dynamic interaction with a given underlying environment. Deep RL techniques have initially been deployed—among many—in the field of gaming [1], robotics [2], personalized recommendations [3], and resource management [4].

In this contribution, we showcase the potential of Deep Reinforcement Learning for problems related to profitable, risk-reduced trading of single-asset financial tools based on historical data (the necessary financial terminology is given in Sect. 1.1). Our starting point is the general—non-finance-related—algorithm developed by Fontaine and Friedman [5] (see also [6, 7]), which is a specific declination of *Multi-Objective* Reinforcement Learning (RL): while we postpone rigorous details to the next sections below, we can—for now—informally describe the methodology in [5] as a RL declination which allows to simultaneously train all possible strategies associated with exploring a dynamic environment. Specifically, each strategy is identified by the reward mechanism associated with a given linear combination of multiple, pre-specified (and possibly conflicting) objective functions. This simultaneous learning process over all possible strategies, which gives the user the freedom to specify the combination of the objective functions after the training has been completed, makes the RL declination in [5] highly interpretable and versatile.

While the techniques in [5] have so far been successfully applied in several fields, applications of such a methodology for financial purposes are—to the best of our knowledge—still lacking. In this paper, we show the potential entailed by using such a Multi-Objective Reinforcement Learning algorithm—along with meaningful variations of the same—in the context of single-asset stock and cryptocurrency applications. We validate our results by deploying the algorithm on several important assets, namely: BTCUSD, ETHUSDT, XRPUSDT, AAPL, SPY, NIFTY50^1^ (see Sect. 4.2). Most importantly, we showcase that reward generalization across multiple objectives in single-asset trading is achievable. In particular, this enables performance gains averaged over all objectives, allowing the trader to switch between different objectives after training. Additionally, we discuss the generalization with respect to the discount factor parameter^2^. We view our proposed methodology as being intuitive and interpretable, having a strong applied connotation, and being complementary to other existing Multi-Objective strategies for financial problems [8–10].

It is worth mentioning that, even though financial problems may be challenging to tackle using AI (this is mostly due to the financial environment’s high data noisiness, subjective definition, and non-reproducibility of time series), Deep RL is widely used in financial applications because it offers the potential to learn complex and adaptive trading strategies directly from unstructured data. Additionally, the use of Deep RL enables the exploration of high-dimensional state and action spaces, as we often find in financial trading where numerous factors influence asset prices.

In the next subsection, we provide the necessary finance-related terminology. Our main results are summarized in Sect. 1.2. The RL-related terminology is instead provided in Sect. 2.

### Single-asset trading: basics

We define single-asset trading as trading limited to buying and selling of individual assets, such as stocks, commodities, indices, or cryptocurrency. This is to be distinguished especially from portfolio trading strategies, which focus on managing multiple assets at once with joint risk assessment.

Input data for machine learning models in single-asset trading include historical price data, market indicators, and relevant contextual information. Typical contextual information is for example, the time of day/week/month, trading volumes at exchanges, and news. The outputs are trading decisions, whether to buy, sell, or hold the asset, and the corresponding quantities or amounts involved in the transactions. For the purposes of this paper, concerning input data, we limit ourself to price data, specifically closing prices, i.e., the price in the last trade of a given day/hour/minute, on a given exchange.

The measure that needs to be maximized or minimized in single-asset trading depends on the specific trading objective. Some examples are maximizing profit, minimizing risk, optimizing a specific performance metric (e.g., Sharpe Ratio or average logarithmic return), or achieving a balance between multiple objectives.

A selection of definitions from the subject area of financial trading:

- *Close price*: The price in the last trade on a given day, on a given exchange.
- *Long position*: The trader holds an amount of the asset *greater* than the sum of their liabilities in the same asset. The trader writes profits when the price rises.
- *Short position*: The trader holds an amount of the asset *less* than the sum of their liabilities in the same asset. The trader writes profits when the price falls.
- *Hold a position*: Neither selling nor buying the asset.

### Contribution of the presented work

We propose a generalized, intrinsically Multi-Objective RL strategy for stock and cryptocurrency trading. We implement this by considering extensions of Multi-Objective Deep Q-Learning RL algorithm with experience replay and target network stabilization given in [5], and deploying it on several important cryptocurrency pairs and stock indexes. We showcase that reward generalization across multiple objectives in single-asset trading is achievable.

Our main findings—which we have validated on several datasets including AAPL, SPY, ETHUSDT, XRPUSDT, BTCUSD, NIFTY50—are described in detail in Sect. 5, and can be summarized as follows.

- *Multi-Reward generalization properties* (cfr. Sect. 5.1). We show that our Multi-Objective RL algorithm generalizes well between four reward mechanisms (last logarithmic return, Sharpe Ratio, average logarithmic return, and a sparse reward triggered by closing positions).
- *Advantage for sparse rewards* (cfr. Sect. 5.2). We show that the results of the Multi-Objective algorithm are significantly better than those of the corresponding Single-Objective algorithm in the case of sparse rewards,^3^ and we provide ablation studies.
- *Discount factor generalization* (cfr. Sect. 5.3). We provide partial evidence of generalization of the discount factor: this parameter is the RL time-regularization parameter in (1).
- *Stability on prediction* (cfr. Sect. 5.4). We use two metrics (Sharpe Ratio and cumulative profits) to show that the prediction of our Multi-Objective algorithm is more stable than the corresponding Single-Objective strategy’s.

Our analysis has been preliminarily conducted in a zero-fee market context (cfr. Sect. 2.3), which is relevant for different kind of traders, specifically: (i) retail traders: zero-fee spot trading is possible on multiple markets, for example, BTCUSD spot on the cryptocurrency exchange Binance, (ii) institutional traders: for them, even more opportunities exist, since they can often operate in low to zero or fixed fee market contexts by directly working together with market makers.

### Structure of the paper

We briefly summarize the state of the art of RL for financial applications and discuss relevant related works in Sect. 2. We provide the abstract setup of our proposed Algorithm, together with the necessary quantitative details, in Sect. 3. After spelling out the main technical features related to our code (see Sect. 4), we discuss our main results in Sect. 5. Conclusions and future outlook are given in Sect. 6.

## State of the art and related work

### Reinforcement learning: basics

#### Single-objective RL

A RL algorithm learns to use the set of observable state variables $\varvec{s}$ (describing the current *state* of the environment) to take the most appropriate admissible *action* *a*. For a general RL algorithm, the state variables and action $(\varvec{s},a)$ and the environment’s intrinsic stochasticity determine the next state of the environment that the algorithm will visit: the exact way in which this task is accomplished varies depending on the specific algorithm. For example, the RL algorithms of so-called *critic–only* form (see [11]) aim to maximize a single, cumulative reward (accounting for all actions taken in a given episode), which is in turn based on a pre-specified state–action *reward function* *r* (assigning a numerical reward $r(\varvec{s},a)$ to every pair $(\varvec{s},a)$ of given state and action undertaken). The algorithm uses several *episodes* (each accounting for an exploration of the environment which ends when a pre-specified end-state is reached) to train its decision-making capabilities (by progressively updating the so-called *Q values* [11] throughout the episodes).

#### Multi-objective RL

Multi-Objective^4^ RL is a declination of RL devoted to learning in environments with vector-valued—rather than scalar—rewards. Such a setting—which allows to cover realistic scenarios in which conflicting metrics are present—has been gaining a lot of traction lately. Three rather comprehensive surveys on the topic^5^ that we are aware of are [12–14]. With methodologies including—among many others—Pareto front-type analysis [15, 16], dynamic multi-criteria average reward reinforcement learning [17], convex hull value iteration [18], Hindsight Experience Replay techniques [19], dynamic weights computation [20], tunable dynamics in agent-based simulation [21], Deep Optimistic Linear Support Learning (DOL) for high-dimensional decision problems [22], Deep Q-networks techniques [23–25], and collaborative agents systems [26], it is safe to say that the Multi-Objective RL paradigm is well established in several non-finance-related applications.

A field which is very closely related to Multi-Objective RL algorithms is the one related to intrinsic motivation approaches. Here, the reward mechanisms are categorized with a finer level of detail. More precisely, the reward mechanisms are not all of the same kind, but rather are split in intrinsic reward mechanisms (these incentivize the RL agent to explore unexplored states of the environment, rather than capitalizing on immediate task reward) and extrinsic reward mechanisms (which do the opposite). This distinction, which has been put to use in different circumstances, is beneficial to many aspects. These aspects include—in addition to enhanced interpretability of the model—balancing of different reward mechanisms based on agents’ preferences (which are expressed through policy votes) [27], learning-adaptive imagination taking also the reliability of the learned dynamics into account [28], and improved balance of algorithm performance/environment exploration and reduced need for reward manual tuning [29].

### Reinforcement learning in finance

A prolific literature for RL in finance is available: the summary report [30] provides an exhaustive overview of the main works associated with the three most commonly used RL paradigms (i.e., *critic*-, *actor*-, and *actor/critic*-based techniques) for financial applications.

#### Single-objective RL in finance

Among critic-based works, we find superiority of RL over standard supervised learning approaches [31], performance improvement assessments with respect to varying reward functions and hyperparameters [32], Deep Q-learning (DQL) extensions to trading systems [33], evolutionary reinforcement learning in the form of genetic algorithms [34, 35], identification of seasonal effects [36, 37], high-frequency analysis [38], trade execution optimization [39], dynamical allocation of high-dimensional assets, [40, 41], and hedging basis risk assessment [42]. For actor-based methods, we mention recurrent reinforcement learning baselines [43, 44], multilayered risk management systems [45], and high-level feature extraction [46, 47]. Finally, we quote [48–50] as representatives of the actor/critic-based category.

#### Multi-objective RL in finance

While the Multi-Objective RL paradigm is well established in several non-finance-related applications (see discussion of Sect. 2.1.2), it appears to still be relatively under-explored^6^ in the context of financial markets.

In this context, the most commonly taken approach to Multi-Objective RL is to indirectly embed the desired Multi-Reward effects in parts of the model other than the reward mechanism itself (e.g., collaborating market agents [8, 51, 52]). Another approach is to consider an intrinsic Multi-Objective approach, but without generalization (i.e., the reward weights are set a priori, and are not part of the learning process). This is the case for the two reference works [9, 10], which we summarize in Sect. 2.3.

Multi-Objective RL has many advantages when applied to real-world decision-making problems in other fields, some examples are given and extensively discussed in [14]. Importantly, these advantages are transferable to financial market applications. In our specific case, we train the agent using multiple profit-risk metrics, each of these metrics represents a distinct approach to handling the ratio between profit-making and risks-taking. In real-world scenarios, financial traders and institutions often change their risk assessments and choice of Key Performance Indicators (KPIs). This can be driven by macro trends, political factors, or any other variables that could not be considered at training-time of the RL agent. Multi-Objective RL makes it possible to adjust the choice and weighting of profit-risk metrics after the training and hence enables traders and institutions to deploy agents to a broader decision-making problem space.

### Related works

The works [9, 10], which are the starting points of the present work, use Multi-Reward scalarization to improve on the following, well-established benchmark strategies in the context of price prediction in single-asset trading: (i) an actor-only, RL algorithm with total portfolio value as Single-Reward, and; (ii) a standard Buy-and-Hold strategy. More specifically, the authors take as rewards the average and standard deviations from the classical definition of the Sharpe Ratio, and combine them with pre-defined weights to favor risk-adjustment. In another variation, the resulting scalarized metric is modified to further penalize negative volatility.

The authors use a two-block LSTM neural network to directly map the last previously taken action (Buy/Sell/Hold) and the available state variables to the next action. The first LSTM block is used for high-level feature extraction, and the other one for reward-oriented decision making. From the experimental results, the authors conclude superiority of their method over to the two benchmarks in terms of cumulative profit and algorithm convergence, although an analysis of statistical significance is not provided.^7^

While the scalarization approach in [9, 10] is effective, it nonetheless has the downside of having to *a priori* specify the balance of the individual rewards (via their weights). This introduces a human factor into the balancing of rewards, and also restricts the scope of the learning process.

Our contribution takes a rather complementary direction with respect to [9, 10]. More precisely:

- We choose not to scalarize the reward metrics, so that the weights can be included in the learning process;
- While the main focus of [9, 10] is increasing the performance of the RL agent given a pre-specified combinations of rewards, our main focus is—instead—always to compare our generalized Multi-Objective RL methodology to a corresponding Single-Objective strategy. Said differently, we do not dwell on increasing the performance of the underlying RL strategy (in fact, we choose a rather simple critic-based RL declination) or on using trading fees (this choice allows us to consider several important trader profiles nonetheless, as discussed in Sect. 1.2). We do this because, in this paper, the real goal is always to evaluate the difference of the Single-/Multi-Objective methods for the same underlying RL method.

#### Remark 1

Given the differences of RL agents and scalarization scope, we do not believe that a *direct* comparison between our work and the works [9, 10] would be fitting. On the other hand, we do see lots of potential for combining and/or bridging between the two approaches in future works. In particular, the adaptation of our methodology with more sophisticated underlying RL methods is deferred to future works.

To the very best of our knowledge, ours is the first application of Multi-Reward RL in the sense of [5] to financial data.

## Proposed approach

We consider variations of the Deep Q-Learning algorithm with Hindsight Experience Replay and target network stabilization [11] (DQN-HER) for both standard Single-Reward or Multi-Reward structure (in the sense of [5]), and apply them to single-asset trading problems.

### The classical setup for (DQN-HER)

The basic structure of (DQN-HER) is concerned with maximizing cumulative rewards of the type

1 $$\begin{aligned} R_t = \sum _{i=t}^{T}{\gamma ^{i}r_i}, \end{aligned}$$

where $\gamma \in (0,1)$ is the so-called discount factor. The discount factor determines the time preference of the agent and regularizes the reward as $T \rightarrow \infty$. A small discount factor makes short-term rewards more favorable. The algorithm fits a neural network taking the current state $\varvec{s}_t$ as input and giving an estimate of the maximum cumulative reward of type (1) achievable by subsequently taking each permitted action $a_t$. The learning process is linked to the Bellman’s equation update

2 $$\begin{aligned}{}[Q(\varvec{s}_t)]_{a_t}&= (1-\alpha )[Q(\varvec{s}_t)]_{a_t} \nonumber \\&\quad + \alpha \left( r(\varvec{s}_t,a_t) + \gamma \max _{a_{t+1}}{[Q(\varvec{s}_{t+1})]_{a_{t+1}}}\right) \end{aligned}$$

for a given learning rate $\alpha \in (0,1)$, and where $r(\varvec{s_t},a_t)$ is the reward for taking action $a_t$ in state $\varvec{s}_t$.

With respect to the previous case, the neural network’s input is augmented by a reward weight vector $\varvec{w}$, which is used to compute the total reward $\varvec{w}\cdot \varvec{r}(\varvec{s}_t,a_t)$ (here $\varvec{r}$ is the vector of rewards, and $\cdot$ denotes the standard scalar product). The Single-Reward case can be seen as a declination of the Multi-Reward case with constant suitable one-hot encoding vectors $\varvec{w}$. The (DQN-HER) algorithm is summarized in Algorithm 1 for the reader’s convenience.
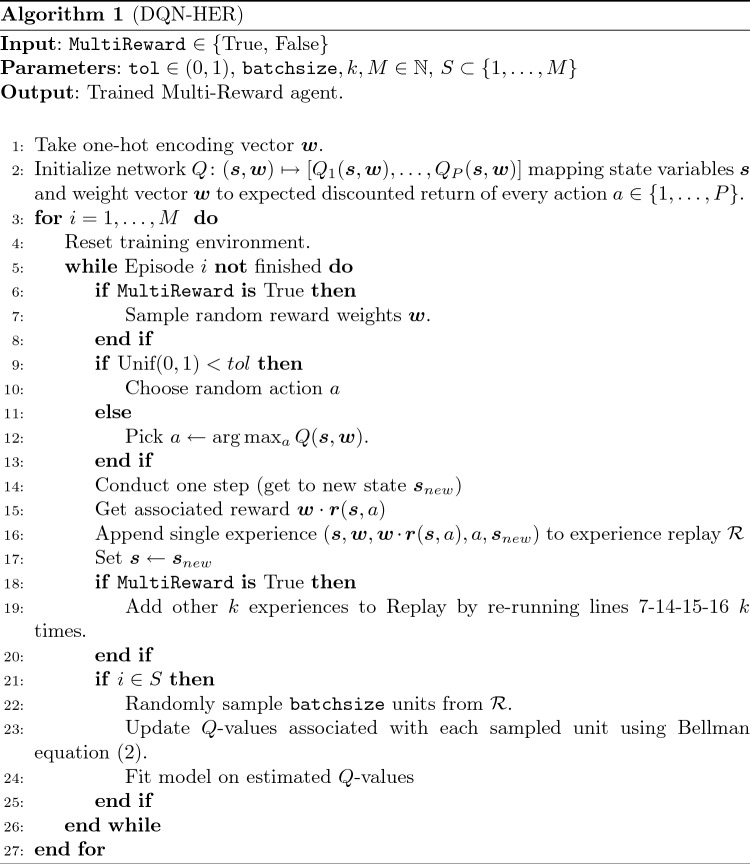


### Our abstract setup

**Fig. 1:**
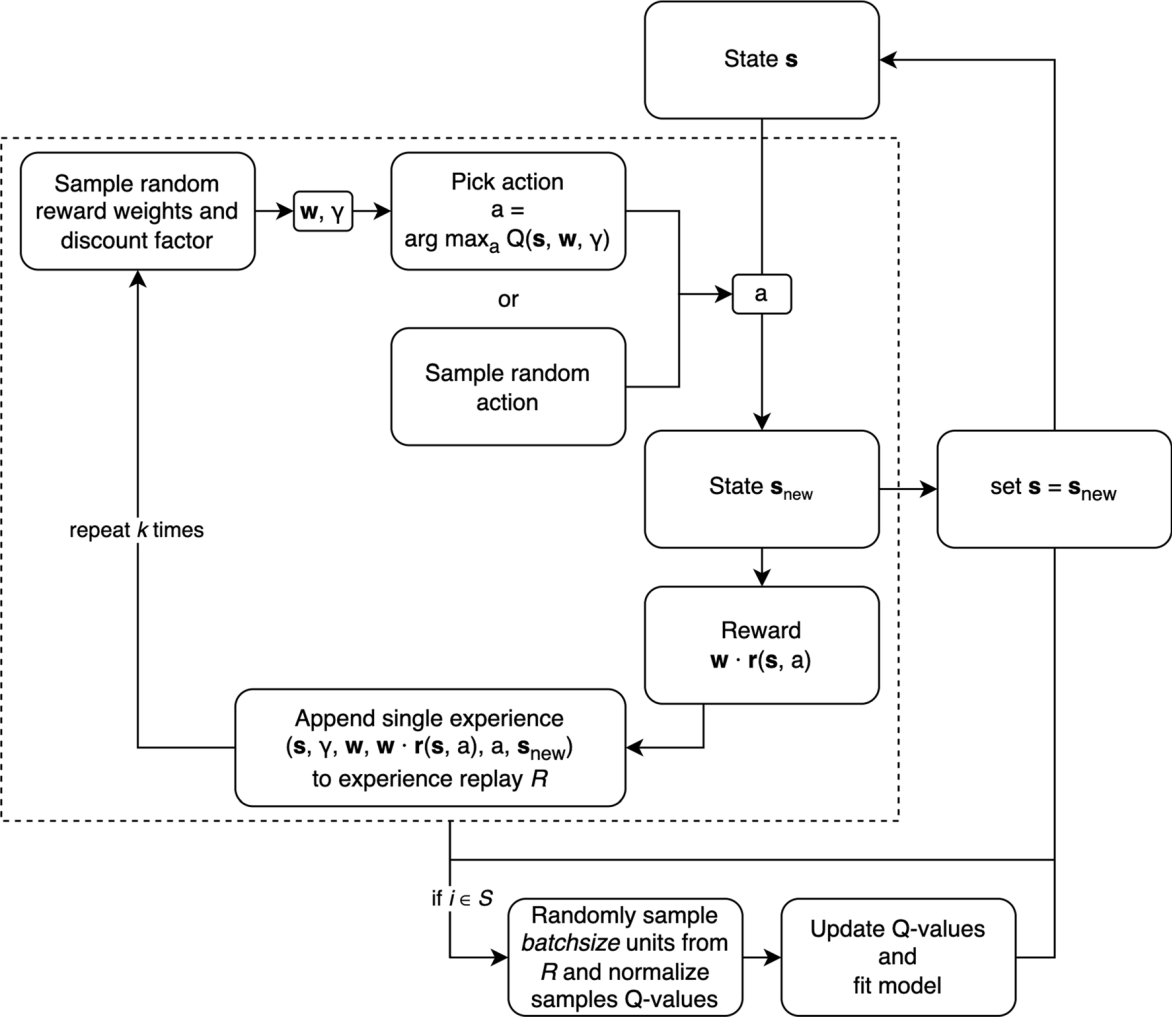
Flow diagram for Algorithm 1

The methodology we adopt in this paper is summarized in Algorithm 2, and stems from the underlying basic Algorithm 1. For the sake of clarity, Algorithm 2 highlights only the changes between the two algorithms. A flow diagram for Algorithm 2 is also provided (cfr. Fig. 1).

These modifications are related to:

(i) an option for ‘random access point’: subject to specification by the user, each training episode may use a subset of the full price history of the training set, where the starting point is randomly sampled and the length is fixed;
(ii) the generalization of the discount factor $\gamma$ as suggested in [5]: this means that the neural network’s input also comprised the discount factor $\gamma$ (i.e., input is augmented from $(\varvec{s},\varvec{w})$ to $(\varvec{s},\varvec{w},\gamma )$);
(iii) the specific choice of normalization spelled out in Sect. 3.2.1.

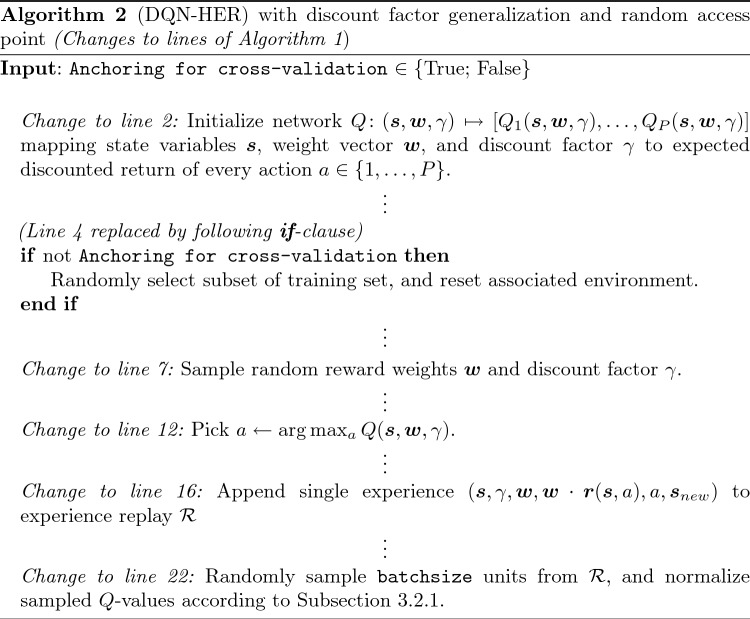

#### Choice of normalization

We choose to indirectly normalize the neural network’s output variables (i.e., the approximate *Q* values) by rescaling the rewards in the Bellman’s update (2). More precisely, whenever a mini-batch

$$\begin{aligned} \{(\varvec{s}_i,\gamma _i,\varvec{w}_i,\varvec{w}_i\cdot \varvec{r}(\varvec{s}_i,a_i),a_i,\varvec{s}_{i, new})\}_{i\in B}, \qquad B\subset \{1,\dots ,\#{\mathcal {R}}\} \end{aligned}$$

is randomly sampled from the replay ${\mathcal {R}}$ (see Algorithm 1, line 12), the rescaled vectors

3 $$\begin{aligned} \displaystyle \tilde{\varvec{r}}(\varvec{s}_i,a_i)&= \frac{\Sigma ^{-1/2} \varvec{r}(\varvec{s}_i,a_i)}{\Vert \varvec{w}_i\Vert _2},\qquad i\in B \end{aligned}$$

and associated scalar rewards $\{\varvec{w}_i\cdot \tilde{\varvec{r}}(\varvec{s}_i,a_i)\}_{i\in B}$ are fed to the training in place of the original sets $\{\varvec{r}(\varvec{s}_i,a_i)\}_{i\in B}$ and $\{\varvec{w}_i\cdot \varvec{r}(\varvec{s}_i,a_i)\}_{i\in B}$. Here, the matrix $\Sigma$ denotes the approximate covariance matrix computed using the reward vectors from the entire replay, namely

$$\begin{aligned} \Sigma = \text{ Cov }\left( \{\varvec{r}(\varvec{s}_i,a_i)\}_{i\in \{1,\dots ,\#{\mathcal {R}}\}}\right) . \end{aligned}$$

With this choice, the overall scalar rewards $\{\varvec{w}_i\cdot \tilde{\varvec{r}}(\varvec{s}_i,a_i)\}_{i\in B}$ are normalized, in the sense that

4 $$\begin{aligned} \text{ Cov }\left( \{\varvec{w}_i\cdot \tilde{\varvec{r}}(\varvec{s}_i,a_i)\}_{i\in B}\right) = 1. \end{aligned}$$

#### Length of the experience replay

We exclusively use a *same-age* type experience replay: more precisely, the experience replay’s oldest element (measured in number of network updates following its creation) is the same for both Single- and Multi-Reward case. In the Multi-Reward case, for every step the RL agent actually takes (Algorithm 1-line 14), a certain number of experiences (*k*) are added to the experience replay ${\mathcal {R}}$, but are not actually visited by the RL agent (Algorithm 1-line 14). Adding unvisited experiences is quite useful, as this enriches the set of experiences on which the RL agent can subsequently be trained on.

### Specific details of our model

After having laid out the general structure of our RL setup (see Algorithm 2), we give precise substance to all quantities involved.

#### State variables $\varvec{s}_t$

We define the state variables $\varvec{s}_t$ as the vector comprising both the current position in the market (which we name $p_t$, and whose precise details are given in Sect. 3.3.2) and a fixed lookback of length $\ell$ (we choose $\ell =24$) over the most recent log returns of close prices $\{z_t\}_t$: more explicitly, we set

5 $$\begin{aligned} \varvec{s}_t := \left( \left\{ \ln z_s - \ln z_{s-1}\right\} _{s = t-(\ell -1)}^{t},\,p_t\right) \in {\mathbb {R}}^{\ell }\times {\mathbb {R}}. \end{aligned}$$

#### Admissible actions and positions

As far as actions are concerned, we analyze two scenarios:

- Long Positions only (LP): The agent is only allowed to perform two actions (Buy/Hold),^8^ and consequently only switch between trading positions Long/Neutral.
- Long and Short Positions (L &SP): the agent is allowed to perform three actions (Buy/Sell/Hold), and consequently switch between trading positions Long/Short/Neutral.

#### Rewards and profit

For a given single-asset dataset with close prices $\{z_t\}_t$, we define the logarithmic (portfolio) return at time *t* as

6 $$\begin{aligned} \ell r_t := \left\{ \begin{array}{rl} \ln z_t - \ln z_{t-1}, &{} \text{ if } \text{ in } {} \texttt{Long} \text{ at } \text{ time } t-1, \\ -\left( \ln z_t - \ln z_{t-1}\right) , &{} \text{ if } \text{ in } {} \texttt{Short} \text{ at } \text{ time } t-1, \\ 0, &{} \text{ if } \text{ in } {} \texttt{Neutral} \text{ at } \text{ time } t-1. \end{array} \right. \end{aligned}$$

Let $L\in {\mathbb {N}}$ be fixed. We focus on three well-established rewards (at a reference given point in time *t*), namely:

(i) the last logarithmic return (LR), which is exactly (6);
(ii) the average logarithmic return (ALR), given by
  $$\begin{aligned} \text{ ALR } := \text{ mean }\left[ \left\{ \ell r_s\right\} _{s=t-(L-1)}^{t}\right] ; \end{aligned}$$
(iii) the non-annualized Sharpe Ratio (SR), given by
  $$\begin{aligned} \text{ SR } := \frac{\text{ mean }\left[ \left\{ \ell r_s\right\} _{s=t-(L-1)}^{t}\right] }{\text{ std }\left[ \left\{ \ell r_s\right\} _{s=t-(L-1)}^{t}\right] }, \end{aligned}$$
  as well as the sparse, less conventional reward:
iv) a ‘profit-only-when-(position)-closed’ (POWC) reward, defined as
  $$\begin{aligned} \text{ POWC } := \left\{ \begin{array}{rl} \ln z_t - \ln z_{t_{LT}}, &{} \text{ if } {} \texttt{Long} \text{ closed } \text{ at } \text{ time } t-1, \\ -\left( \ln z_t - \ln z_{t_{LT}}\right) , &{} \text{ if } {} \texttt{Short} \text{ closed } \text{ at } \text{ time } t-1,\\ 0, &{} \text{ otherwise }, \end{array} \right. \end{aligned}$$
  where $t_{LT}$ is the time of last trade (i.e., last position change).

## Experiments

This section provides a detailed description of the experimental setup used to validate the approach suggested in Sect. 3. This includes the software components employed, measures taken to ensure reproducibility, and the dataset used for experimentation.

### Codebase

For the structure of our code, we took some inspiration from two open-source repositories: the FOREX and stock environment at

https://github.com/AminHP/gym-anytrading

and the minimal Deep Q-Learning implementation at

https://github.com/mswang12/minDQN .

#### Open-source directory and reproducibility

Our entire code is provided in open-source format at

https://github.com/trality/fire .

In particular: the instructions for reproducibility are contained in the README.md file therein; we provide the entirety of the datasets considered in our experiments.

### Datasets

We perform several runs of experiments on a variety of relevant single-asset datasets, both in cryptocurrency and stock trading.

In the interest of increasing the training capabilities of our experiments (see Sect. 5.4), we always include an evaluation set in addition to train and test sets. The percentages of the data associated with training/evaluation/test sets are roughly *train*:64%–*eval*:16%–*test*:20%. All datasets are of sufficient length as to provide a reasonable compromise between experiment running times, and significance of predictions.

#### Cryptocurrency pairs

We consider the following datasets: *hourly*-close price-data points for the BTCUSD pair (August 2017–June 2020); *hourly*-close price-data points for the ETHUSDT pair (August 2017–June 2020); *hourly*-close price-data points for the XRPUSDT pair (May 2018–March 2021).

#### Traditional capital market

We consider three datasets: (1) The NIFTY50 index, which is a weighted average of 50 of the largest Indian companies listed on the National Stock Exchange. (2) The SPY exchange-traded fund, which tracks the S &P 500 stock market index. (3) AAPL, the stock price of Apple Inc. as traded on the Nasdaq stock exchange. The date range for AAPL, SPY is January 2000–September 2022 (*daily*-close price-data points), while for NIFTY50 is March 2020–June 2020 (*minute*-close price-data points).

**Fig. 2:**
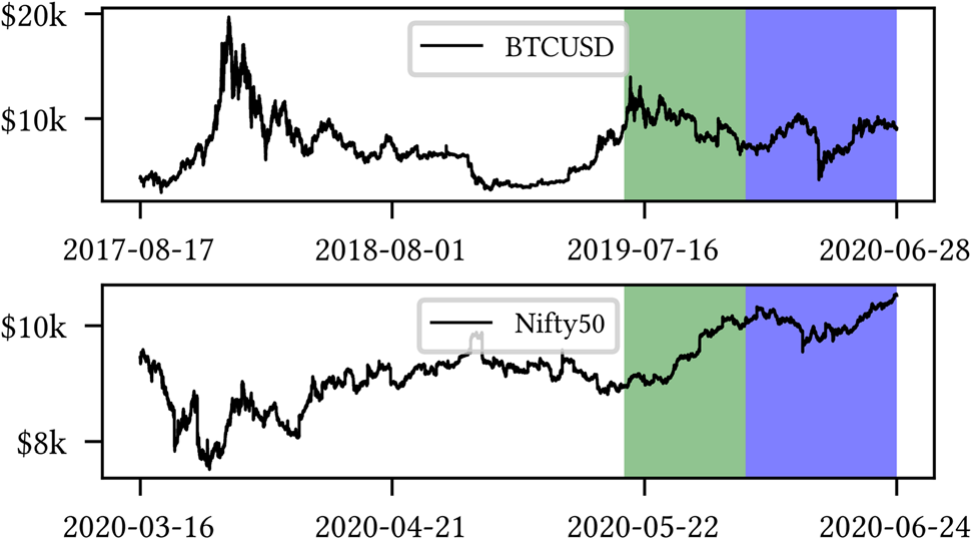
Close prices for train (white), evaluation (green), and test set (blue). Top (*Bottom*): BTCUSD (*NIFTY50*). We notice the mostly downward trend (respectively, *strongly* upward trend) on the BTCUSD (*NIFTY50*) evaluation set (colour figure online)

A snapshot example from the datasets BTCUSD and NIFTY50 is shown in Fig. 2.

### Quantities of interest and benchmarks

All our considerations will be based on the following—quite standard—quantities:

- Total Reward: the cumulative reward over the considered portion of the dataset.
- Total Profit: the cumulative gain/loss obtained by buying or selling with all the available capital at every trade.
- Sharpe Ratio: the average return per step, divided by the standard deviation of all returns.

Crucially, results of Multi- and Single-Reward simulations are compared against each other, as well as—individually—also against the Buy-and-Hold strategy.

The Sharpe Ratio is a widely accepted measurement for performance of financial trading strategies. In this work, we adopt the Sharpe Ratio as an indirect measurement of our RL agents performance.

### Validation of results

In order to show the robustness of our analysis, we adhere to the following general strategy.

- $\bullet$ *Large number of datasets*. We run our experiments on a large group of single-asset financial time series: in particular, these include cryptocurrency pairs (BTCUSD, ETHUSDT, XRPUSDT) and stock indexes (AAPL, SPY, NIFTY50).
- $\bullet$ *Different initializations*. In order to detach the impact of the chosen neural network’s random initialization from the actual results, we perform several independent trainings for each given dataset we run our algorithm on. This is used to assess the distribution of gains for our algorithm.
- $\bullet$ *Cross-validation*. We further confirm the effectiveness of our method using a plain hold-out method and *walk-forward* cross-validation with anchoring (i.e., where the training set starting date is the same for all folds).

### Measures for code efficiency

#### Basic measures

The most important measures taken in this regard are as follows. Firstly, as we are primarily interested in assessing the potential superiority of a Multi-Reward approach over a Single-Reward one (see discussion in Sect. 2.3), we decide to stick to a simple multi-layer perceptron (MLP) neural network (Algorithm 1-line 2). Secondly, for the purpose of checking the performance in between training, we run the currently available model on full training and evaluation sets only for an evenly distributed subspace of episodes.

#### Option of random access point

If we choose the walk-forward cross-validation, the training in each episode is performed on a randomly selected, contiguous subset of the full training set with pre-specified length (this reduces the overall training cost).

#### Vectorized computation of *Q* values

Except for the trading position $p_t$, the time evolution of the state variables vector given in (5) is otherwise entirely predictable (as prices $z_t$ obviously do not change in between training episodes). This implies that, given a predetermined set of *n* actions, the algorithm can efficiently vectorize the evaluation of the neural network for each separate trading position, and then deploy the results to speedily compute the associated *n* future steps. This method is feasible as the cardinality of admissible values of non-predictable state variables (i.e., the trading position) is low (three at most, in the L &SP case).

## Results

**Fig. 3:**
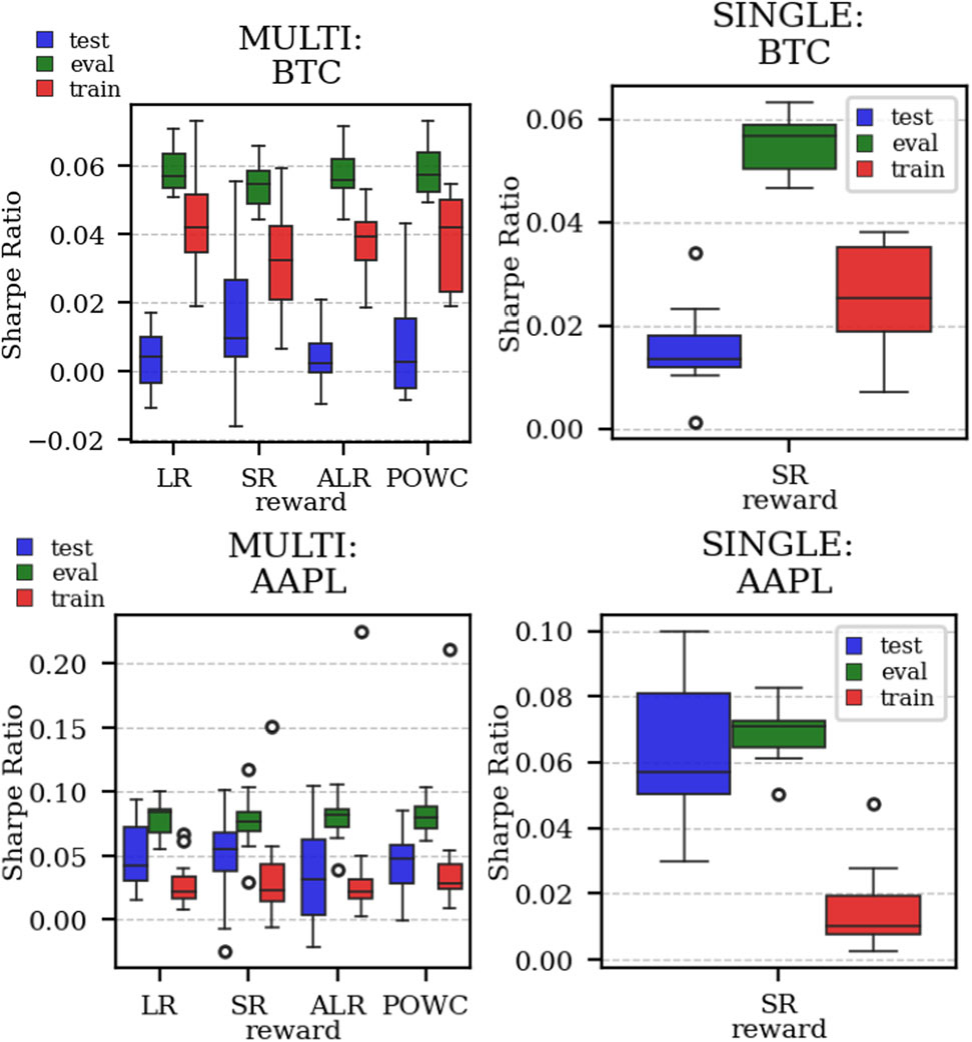
Distribution of the performance of multiple experiments with different random initialization for BTCUSD and AAPL on training, evaluation, and test datasets, with Multi-Reward and Single-Reward

The results arising from several Multi- and Single-Reward experiments (using Algorithm 2) on several datasets (BTCUSD, ETHUSDT, XRPUSDT, AAPL, SPY, NIFTY50) give us four general indications (as detailed in Sect. 1.2), which we discuss below in detail in as many dedicated (Sects. 5.1, 5.2, 5.3, 5.4). We support our analysis using different types of plots, namely:

- (i) distribution of performance (box-plots for various rewards over train/eval/test sets) obtained using several independent experiment realizations, see Fig. 3 as an example; the average number of independent experiment realizations for these plots is around ten.
- (ii) cumulative rewards on train/eval sets (as functions of epochs), see Fig. 14 as an example, and;
- (iii) for each epoch, the Sharpe Ratio on eval set of the current best model, see Fig. 9 as an example.

### Multi-reward generalization properties

The first crucial conclusion that we can comfortably jump to is that the Multi-Reward strategy generalizes well over all different rewards, and this can be seen on pretty much all plots which compare Multi-Reward and Single-Reward.

**Fig. 4:**
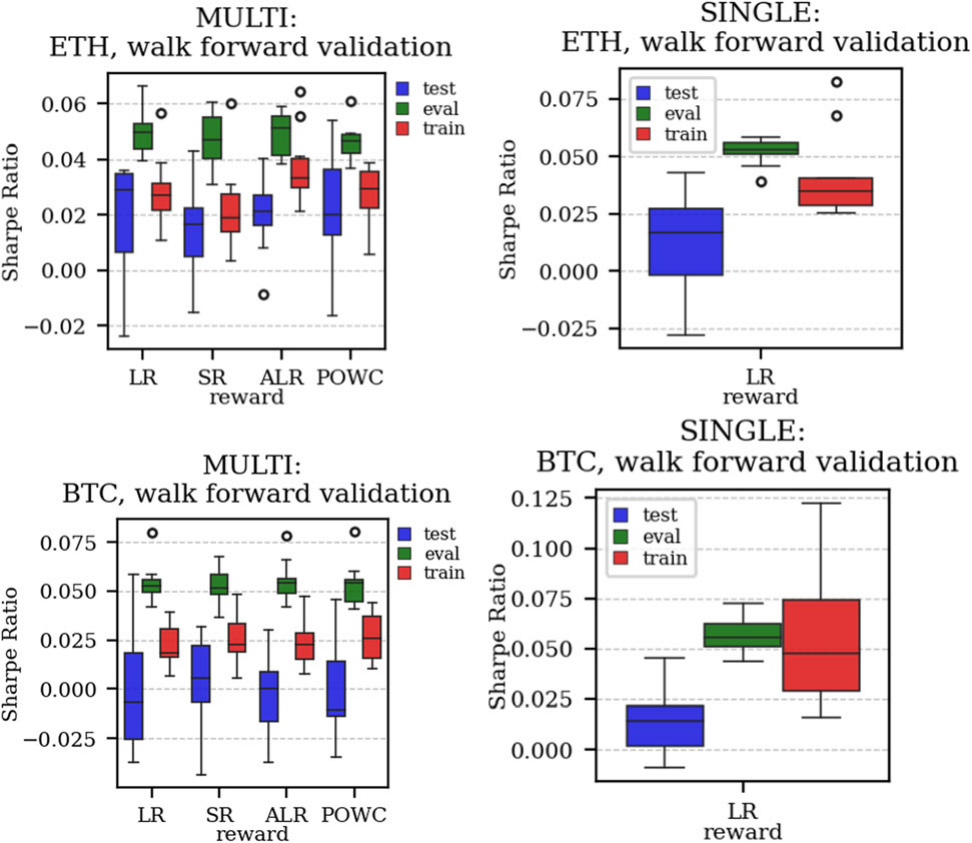
Distribution of the performance of multiple experiments with different random initialization. Results are shown for ETHUSDT and BTCUSD on training, evaluation, and test datasets. We compare Multi-Reward and Single-Reward using anchored walk-forward validation

**Fig. 5:**
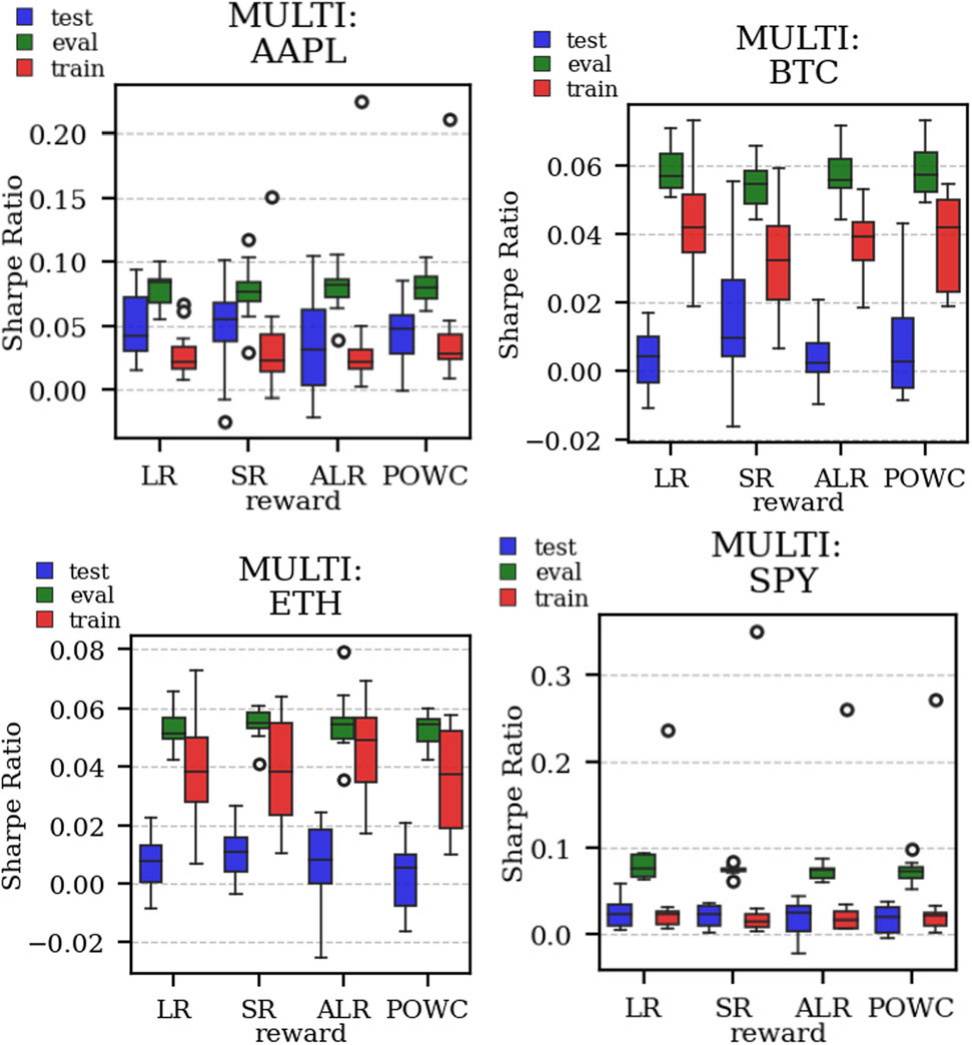
Distribution of the performance of multiple experiments with different random initialization for different assets on training, evaluation, and test datasets, with Multi-Reward

**Fig. 6:**
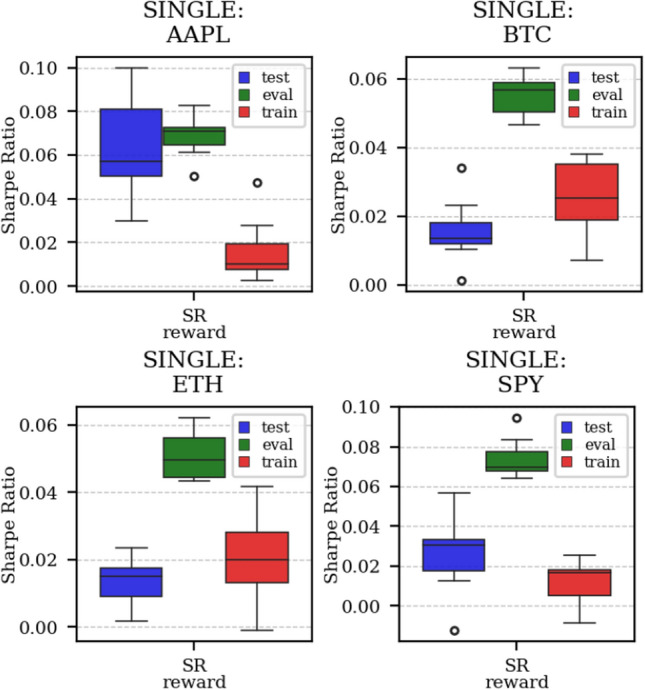
Distribution of the performance of multiple experiments with different random initialization for different assets on training, evaluation, and test datasets, with Single-Reward (SR)

Firstly, the generalization can be observed in average terms, for instance, in Fig. 3 (Single vs Multi for SR metric on AAPL and BTCUSD), Fig. 4 (Single vs Multi for LR metric on ETHUSDT and BTCUSD with walk-forward validation) and from the comparison of Figs. 5 and 6.

**Fig. 7:**
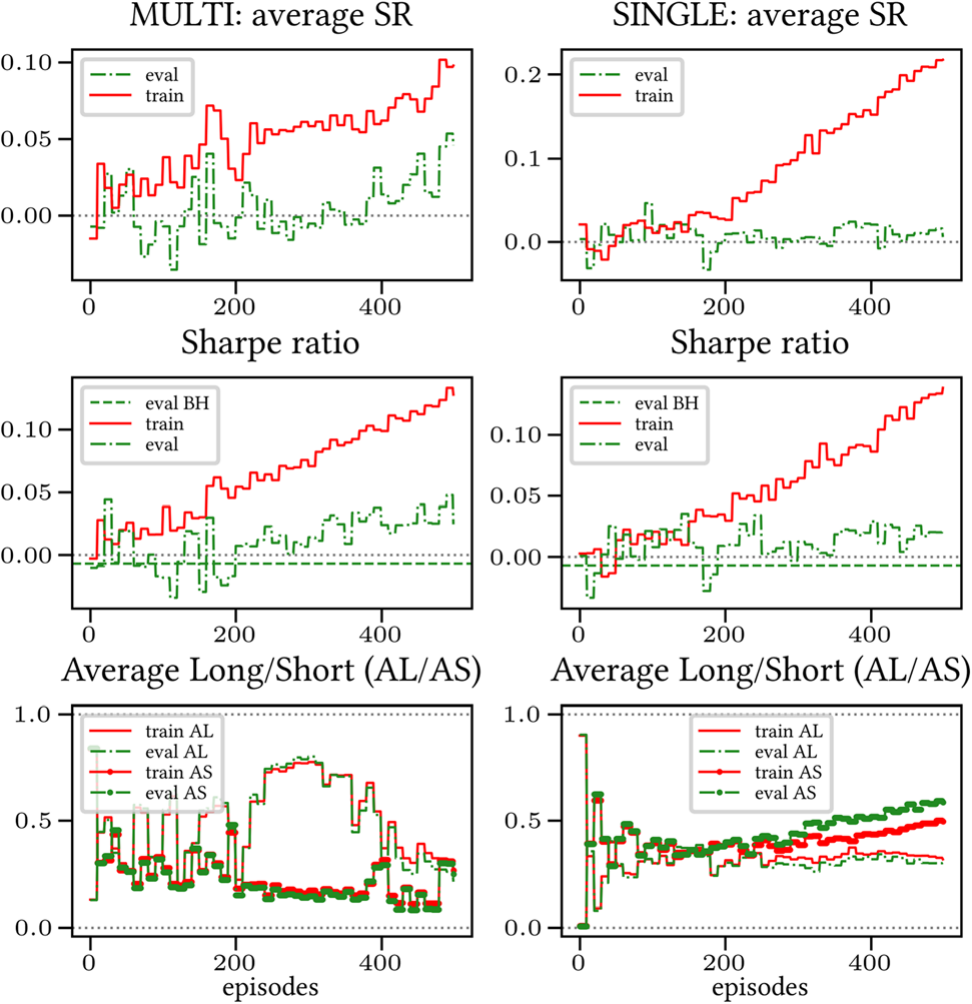
Reward Performances, SR/L &SP/BTCUSDT, regularized agent’s network

**Fig. 8:**
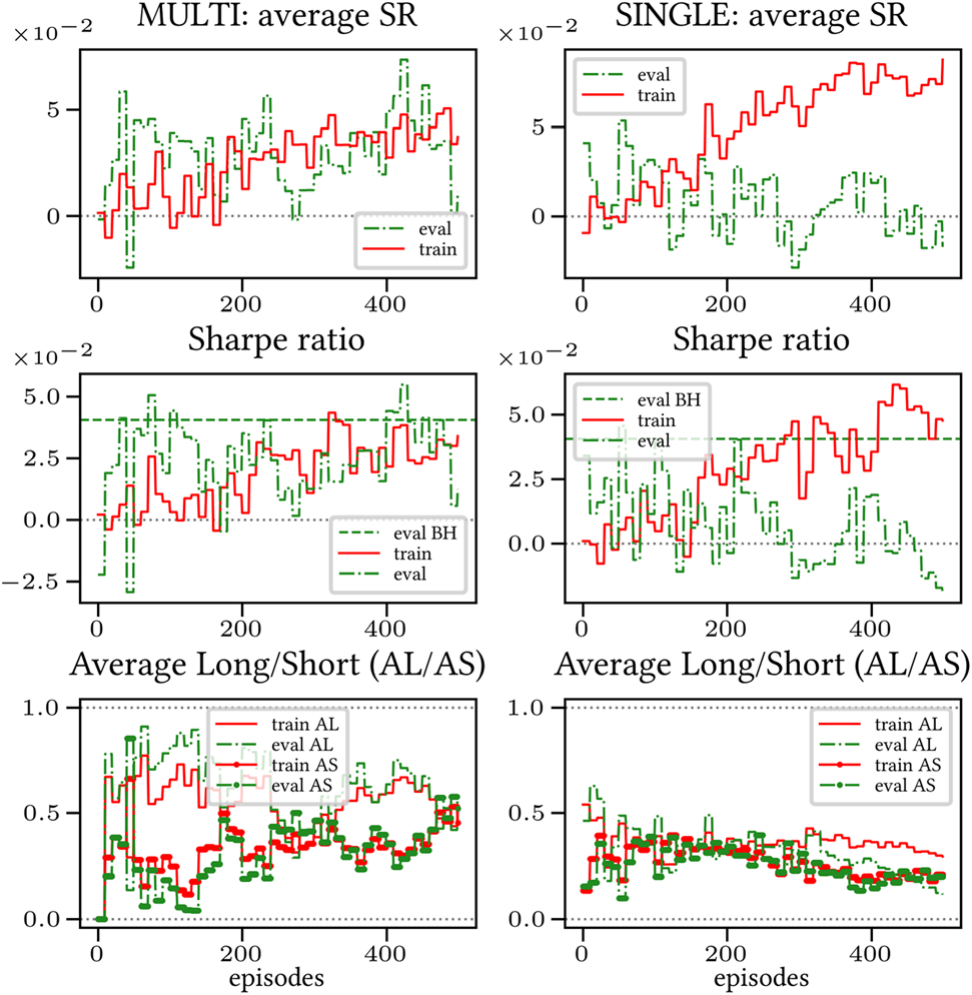
Reward Performances, ALR/L &SP/NIFTY50, regularized agent’s network

**Fig. 9:**
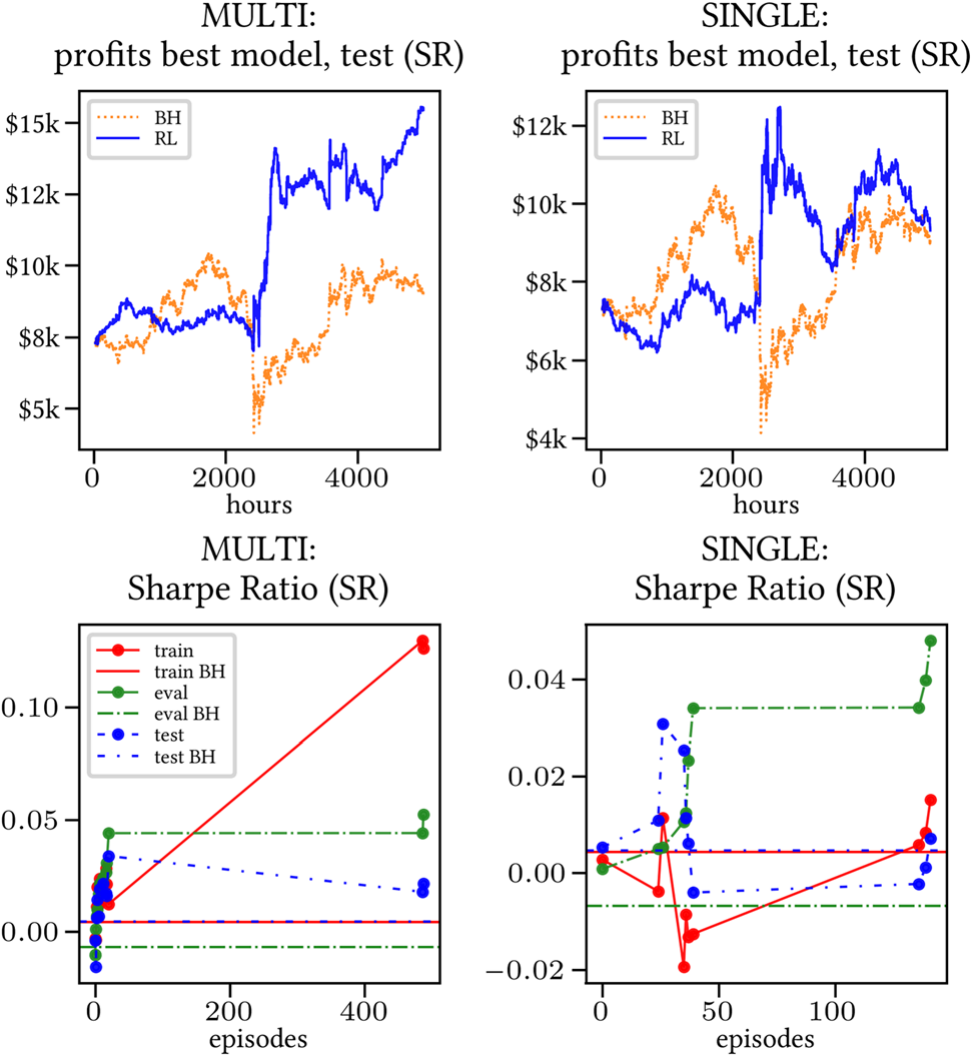
Top (*Bottom*): Best model for profits (*performance for training/evaluation/test set based on SR*), BTCUSD

**Fig. 10:**
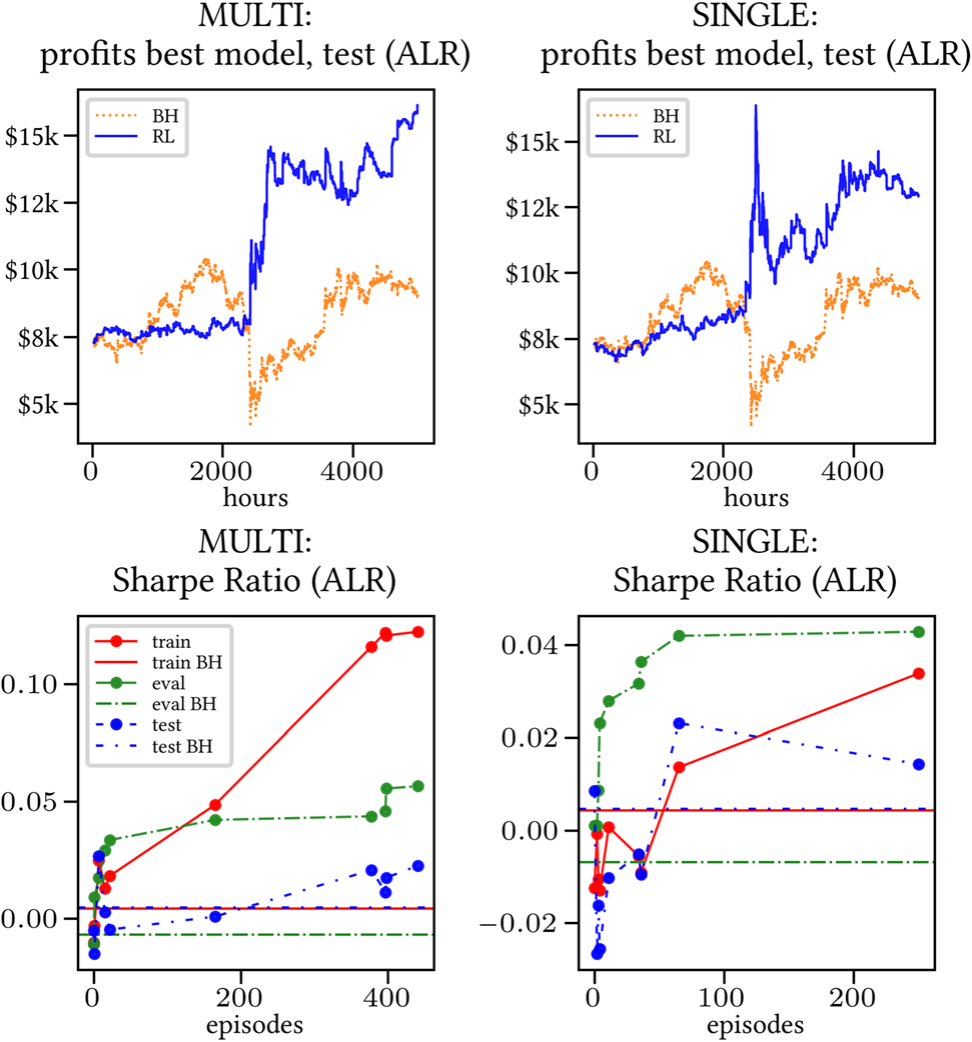
Top (*Bottom*): Best model for profits (*performance for training/evaluation/test set based on ALR*), BTCUSD

**Fig. 11:**
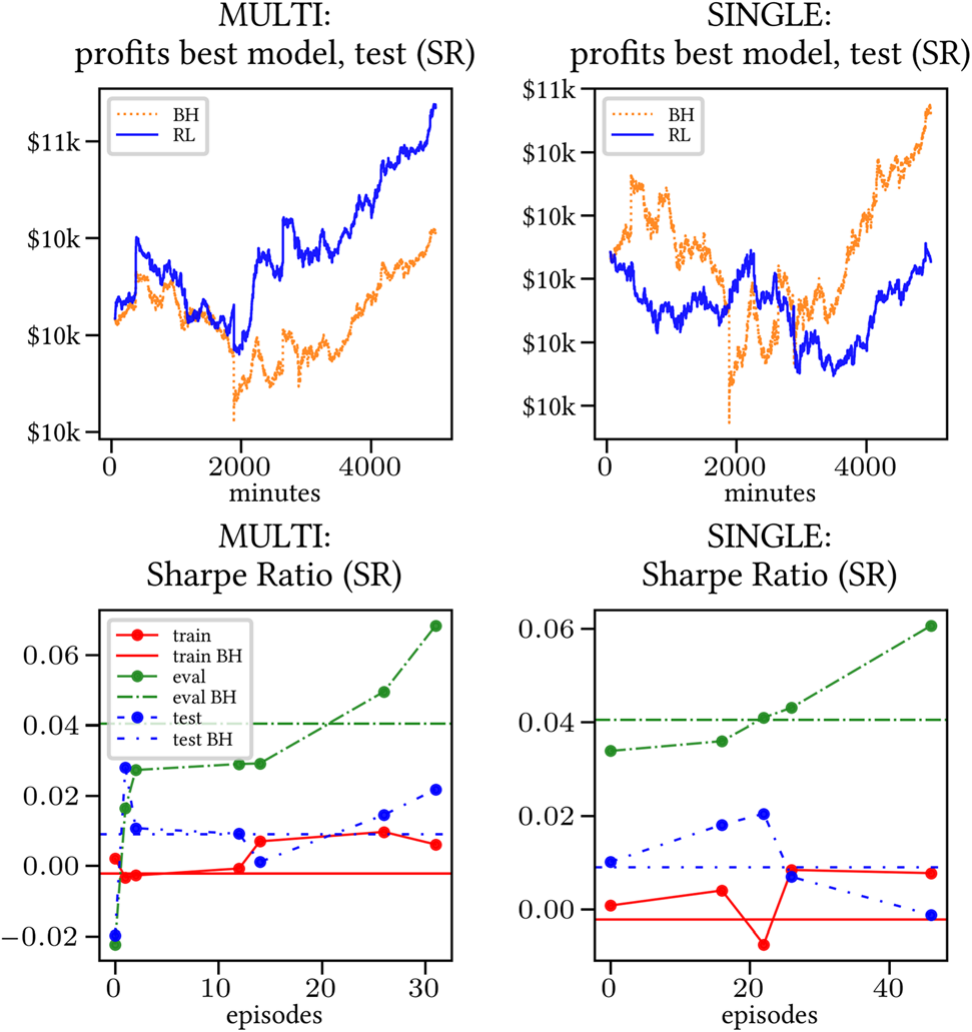
Top (*Bottom*): Best model for profits (*performance for training/evaluation/test set based on SR*), NIFTY50

Secondly, the generalization is also visible for cumulative rewards in Figs. 7 and 8 (Single vs Multi for SR metric on BTCUSD and ALR metric on NIFTY50). Thirdly, as far as the predictive power is concerned, the Multi-Reward method is as performing as—and sometimes better performing than—the Single-Reward counterpart, see Figs. 9, 10 and 11.

#### Remark 2

The training saturation levels may differ from those of the corresponding Single-Reward simulations, although this is likely caused by an apparent regularization effect of the Multi-Reward setting.

#### Remark 3

**Fig. 12:**
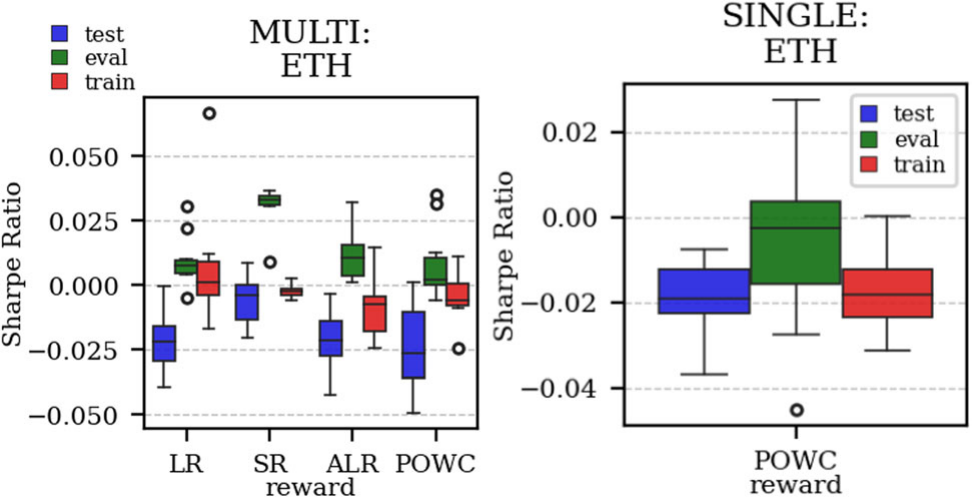
Distribution of the performance of multiple experiments with different random initialization for ETHUSDT on training, evaluation, and test datasets, with Multi-Reward and Single-Reward (POWC) including fees of $0.03\%$

The performance in the case of non-null fees (see Fig. 12) is poor: this is not related to the generalization (which seems to hold also in this case), but rather to the simple nature of the underlying RL algorithm, see discussion in Sect. 2.3.

**Fig. 13:**
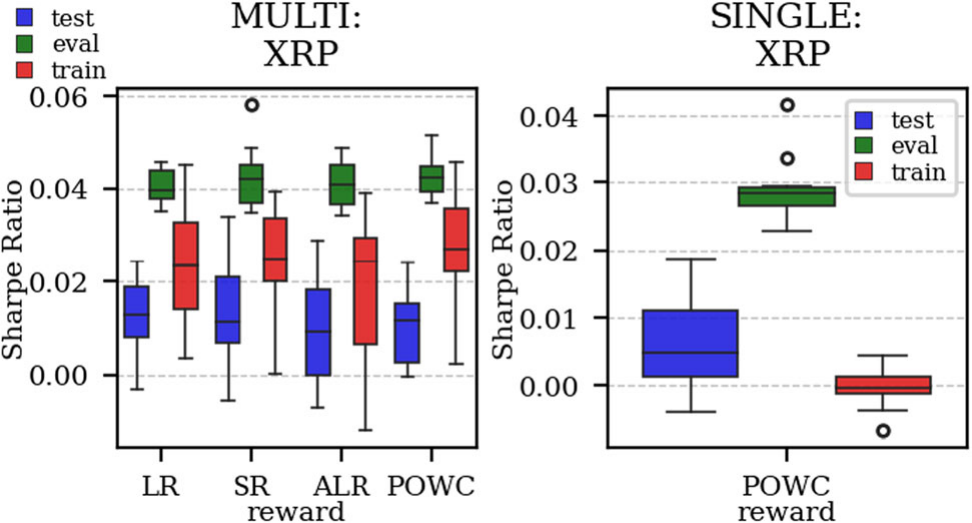
LP case: Distribution of the performance of multiple experiments with different random initialization for XRPUSDT and POWC on training, evaluation, and test datasets, with Multi-Reward and Single-Reward

#### Practical multi-reward advantage

As discussed in Sect. 2.2.2, Multi-Reward training offers more flexibility for agent adjustments after the training-phase. The gain in flexibility however is only worth if we do not experience a decrease in performance. In our experiments, we observed no performance decrease comparing the Single-Reward approach with the corresponding Multi-Reward approach. For instance, the POWC Single-Reward in Fig. 13 on the right and corresponding the POWC of the Multi-Reward trained agent on the left show similar performance. Specifically, the majority of the Single-Reward experiments scored a POWC performance between 0.001 and 0.011, and the majority of the Multi-Reward experiments had a POWC performance between 0.003 and 0.015.

To illustrate the practical implications, we consider a real-world scenario in which a trader or institution previously adopted the POWC as their main KPI and later (after the agents training-phase) transitions to using the Sharpe Ratio (SR). In Fig. 13, we observe that the Multi-Reward trained agent outperformance the Single-Reward trained agent when the reward (SR) is chosen corresponding to the performance metric (Sharpe Ratio (SR)). The majority of Multi-Reward experiments scored a SR performance between 0.007 and 0.021. This implies that the Multi-Reward approach can be adapted more effectively to new objectives, such as switching from POWC to SR, resulting in improved performance.

### Multi-reward improvement on strongly position-dependent rewards

Let us consider a trading reward which

- (i) is strongly dependent on a specific trading position, and;
- (ii) is *sparse* (meaning that it might take several time steps for such a reward to return a nonzero value).

Intuition says that it is highly likely that the Single-Reward RL algorithm will struggle to learn based on such a reward. On the other hand, it is expected that a Multi-Reward algorithm will perform better, due to the influence of easier rewards with different but similar goals. Furthermore, the performance difference between Multi-Objective and Single-Objective method is expected to be even more pronounced when there are fewer trading positions allowed (thus further restricting the Single-case capabilities to learn).

Below, we confirm these intuitions for the POWC reward—which satisfies (i) and (ii) above—by running the Multi-Reward RL code with all four rewards considered in Sect. 3.3.3 in its dictionary.

#### Case LP

**Fig. 14:**
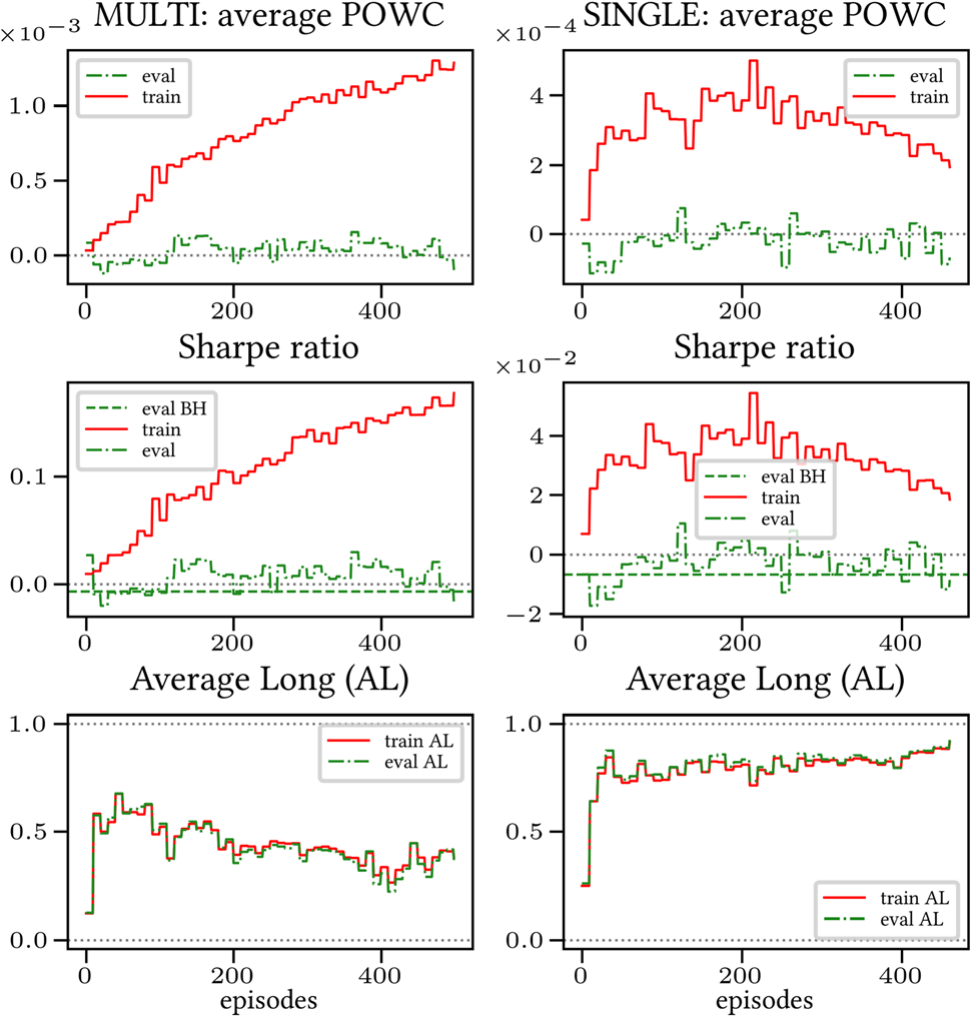
Reward performances, POWC/LP/BTCUSDT

**Fig. 15:**
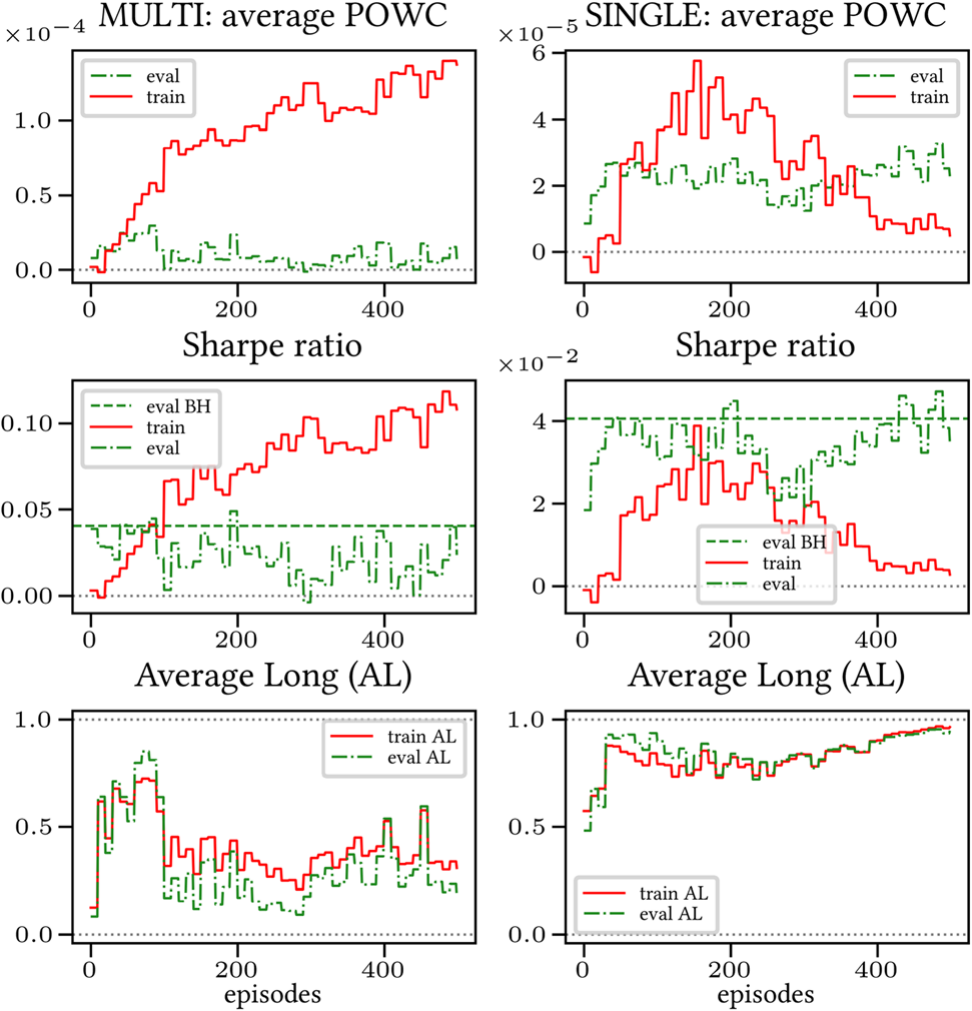
Reward performances, POWC/LP/NIFTY50

**Fig. 16:**
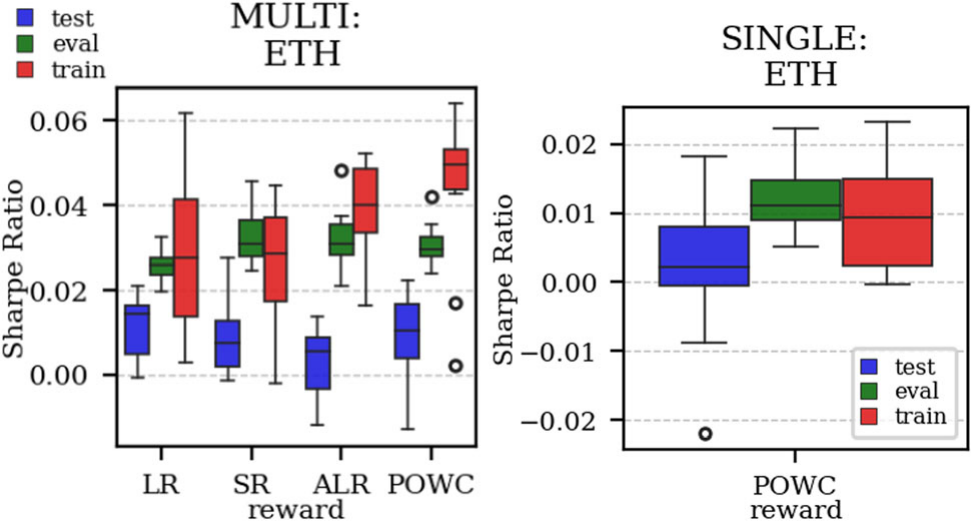
LP case: Distribution of the performance of multiple experiments with different random initialization for ETHUSDT and POWC on training, evaluation, and test datasets, with Multi- and Single-Reward

When opening Short positions is not allowed, the POWC reward provides a nonzero feedback only when Long positions are closed. This extremely sparse feedback is likely to be the justification of the poor cumulative training performance (Figs. 14 and 15) where Single-Reward saturates the training at a much lower level than Multi-Reward. In contrast, the training for the Multi-case algorithm is much more consistent, as it can benefit from rewards with similar goals as POWC, but with more frequent feedback (e.g., LR). In the Multi-Reward case, the prediction performance exceeds the Buy-and-Hold threshold on the BTCUSD dataset in a more consistent and stable way than in the training saturation regime of the Single-Reward case. Furthermore, the average of Long positions^9^ is lower (which means less risk is taken) and are also relatively stable. The difference in strategy between Multi- and Single-Reward algorithm can be inferred by the different convergence of Long Positions. The analysis is further consolidated by considering the stark contrast in saturation levels in the distributional plots for the XRPUSDT and ETHUSDT, see Figs. 13 and 16.

#### Case L &SP

**Fig. 17:**
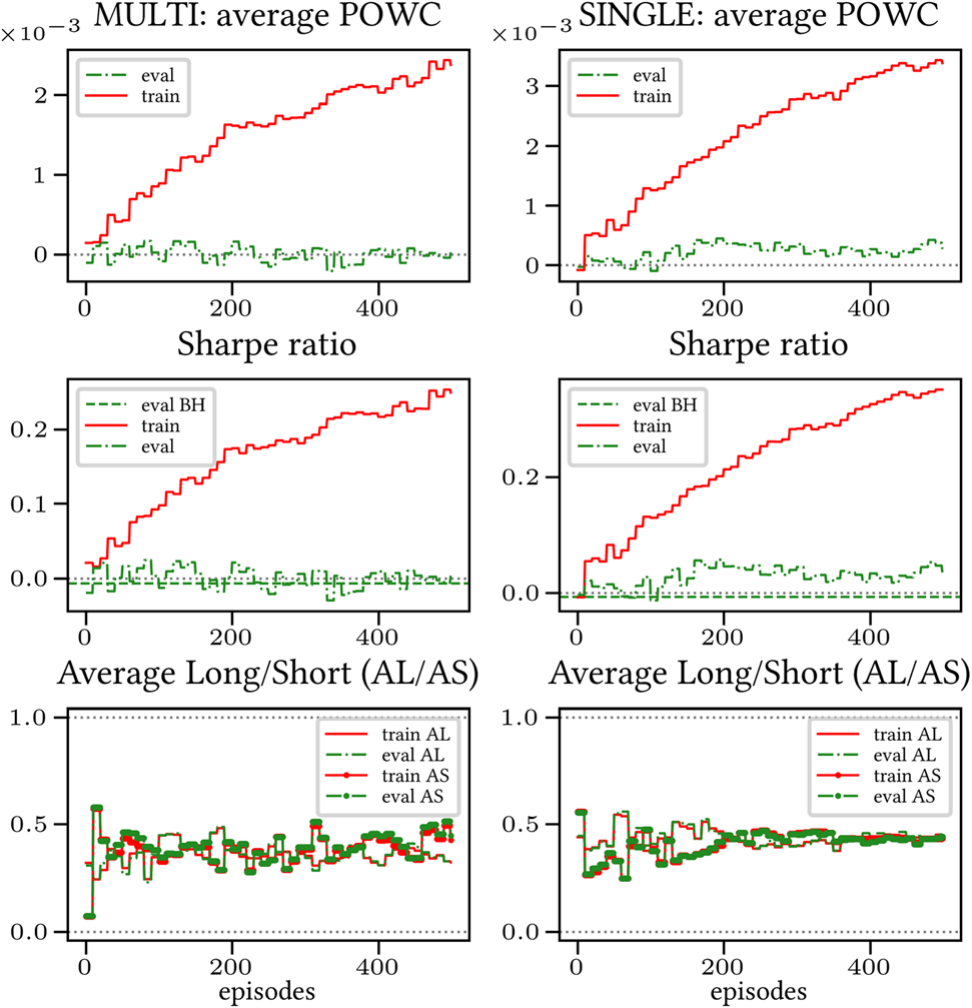
Reward performances, POWC/L &SP/BTCUSDT

**Fig. 18:**
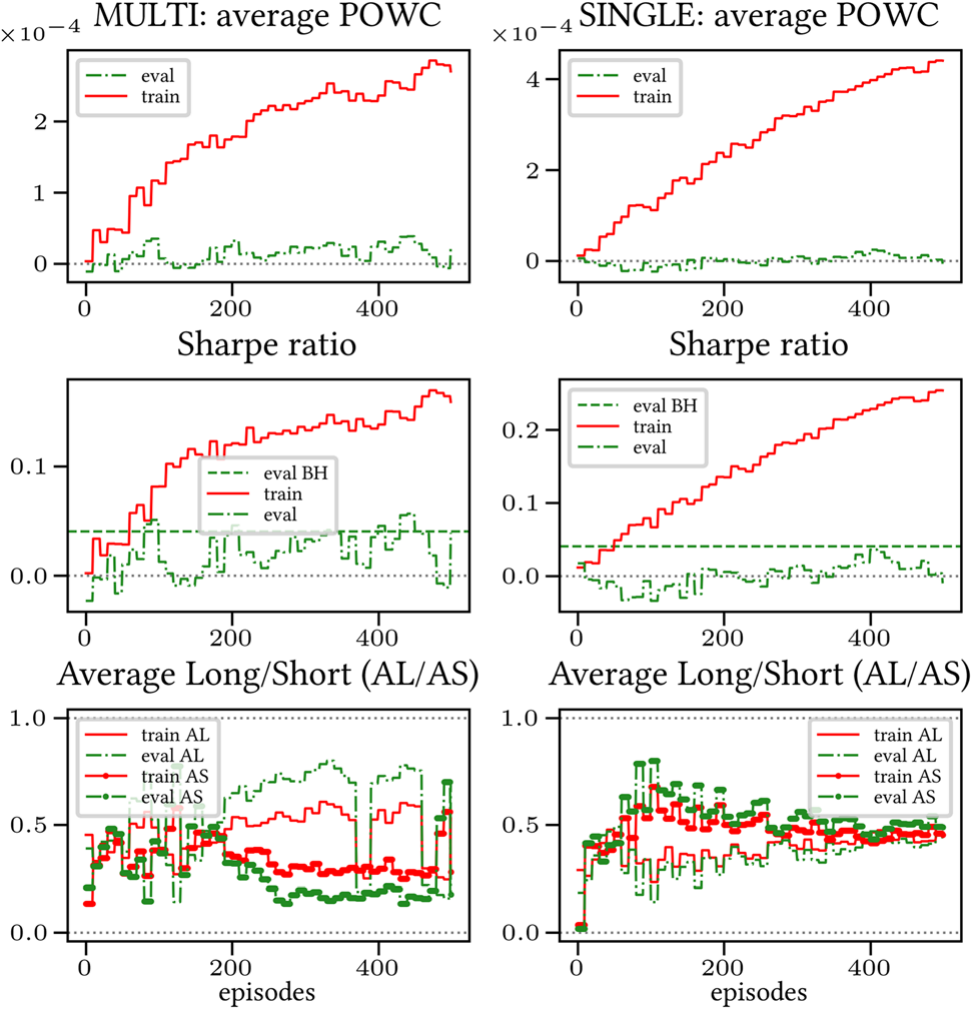
Reward performances, POWC/L &SP/NIFTY50

**Fig. 19:**
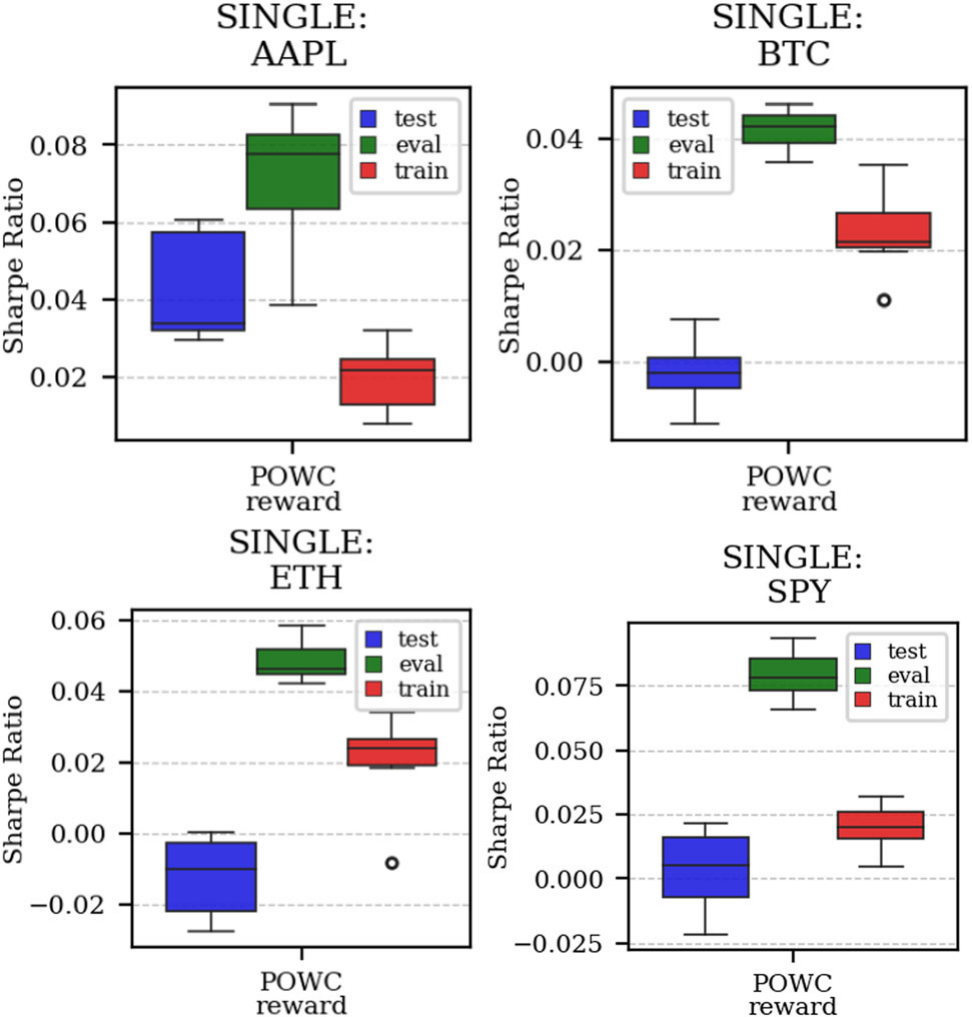
Distribution of the performance of multiple experiments (7) with different random initialization for different assets on training, evaluation, and test datasets, with Single-Reward (POWC). For ETHUSDT, notice the difference in performance with the case with only Long positions allowed (see Fig. 16)

As expected, the difference between multi- and single case is much narrower in this case, as Short positions can now be opened, see Figs. 17 and 18. Except for the training saturation, relevant differences are not noted. This is also visible in distributional plots (for instance, consider the SPY pair in Figs. 5 and 19).

#### Ablation studies for ETHUSDT dataset

**Fig. 20:**
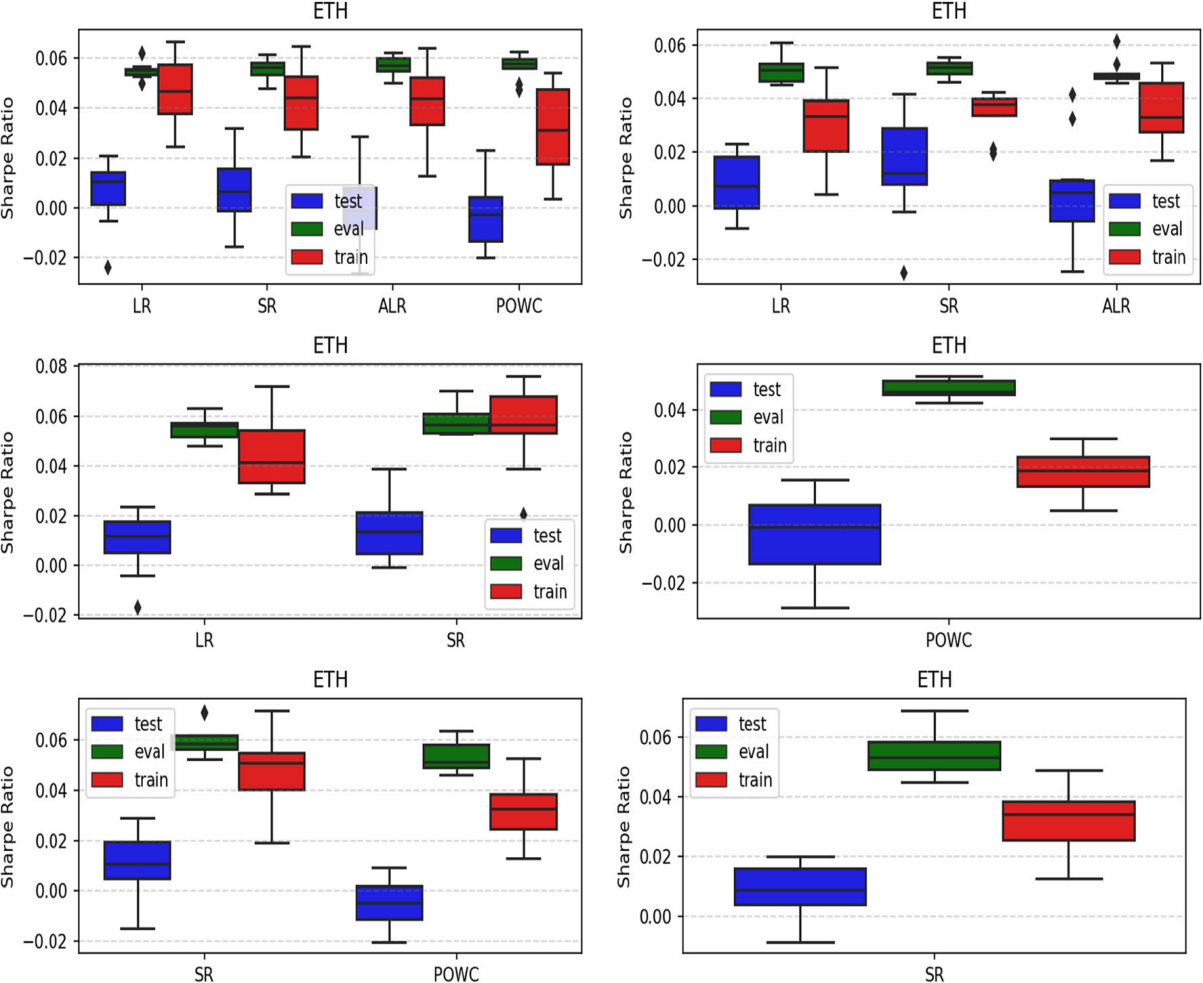
Ablation studies for ETHUSDT dataset, for different combinations of rewards

The discussions held in the previous two tions is part of a broader question, specifically: which metrics (if any) have the greatest positive influence on our generalization procedure? In order to answer this, we have performed ablation studies, meaning that we have run several experiments, each one of them with a different subject of metrics (on the dataset ETHUSDT only). The results can be seen in Fig. 20.

Our conclusion is twofold:

- (i) the SR metric appears to have the biggest influence on performance. This fact (which is not surprising, as SR is the most robust financial metric of the four) can be seen in the comparison between using up for metrics and only using LR/SR (Fig. 20, top-left vs center-left), by the fact that POWC is positively influenced by SR alone (Fig. 20, center-right vs bottom-left) and that such a positive influence is not significantly altered if the other metrics are added (Fig. 20, center-right vs top-left).
- (ii) SR is often positively influenced by other—weaker—metrics. This fact is more surprising, and rather interesting. This fact can be seen on the improved test/train level of SR when paired with the—weakest of all—POWC (Fig. 20, bottom-left vs bottom-right), the improved train level of SR when paired with LR/ALR (Fig. 20, top-left vs bottom-right), and the improved train level of SR when paired with LR (Fig. 20, center-left vs bottom-right).

While fact (i) is reasonably predictable, and good in and of itself, fact (ii) is even more important, as it shows that even consolidated financial metrics can benefit from our Multi-Objective method when they are paired with metrics that are—on paper—supposedly less effective.

### Random discount factor generalization

**Fig. 21:**
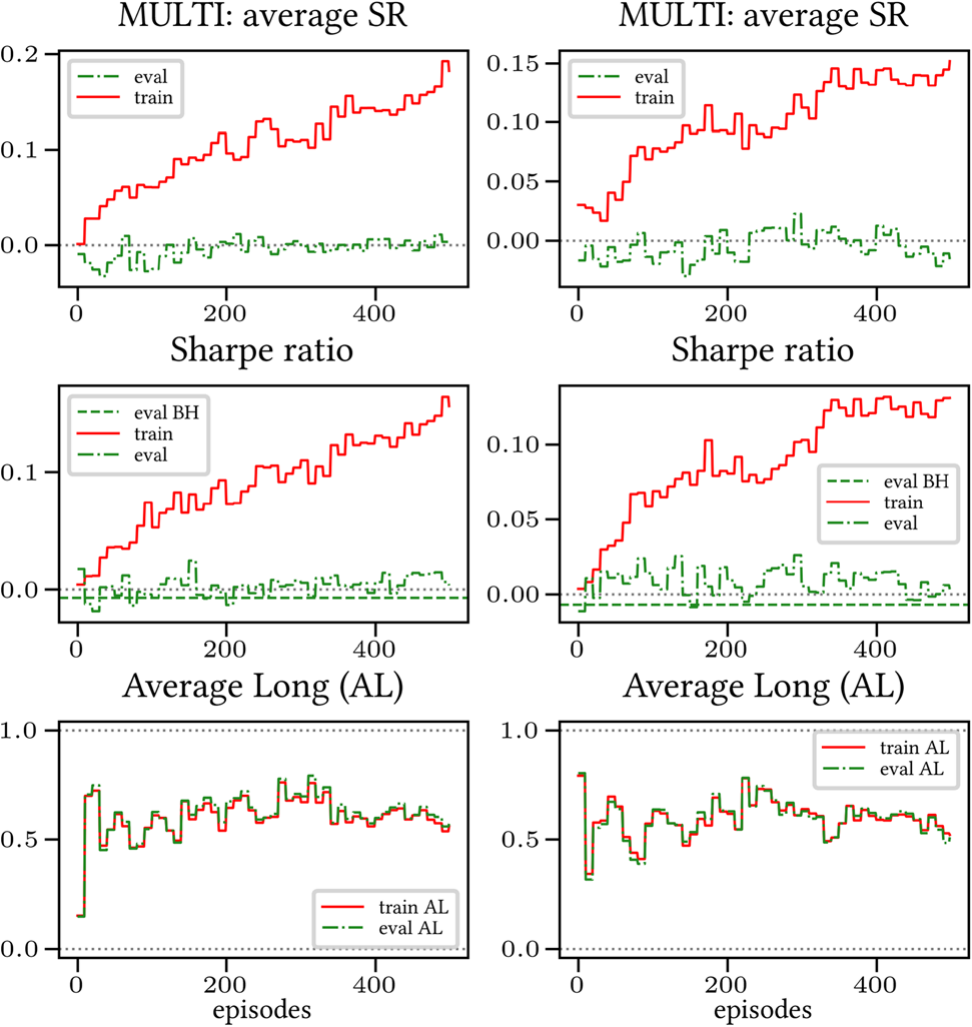
Reward Performances, SR/LP/BTCUSDT. Left (*Right*): Non-random (*Random*) discount factor

**Fig. 22:**
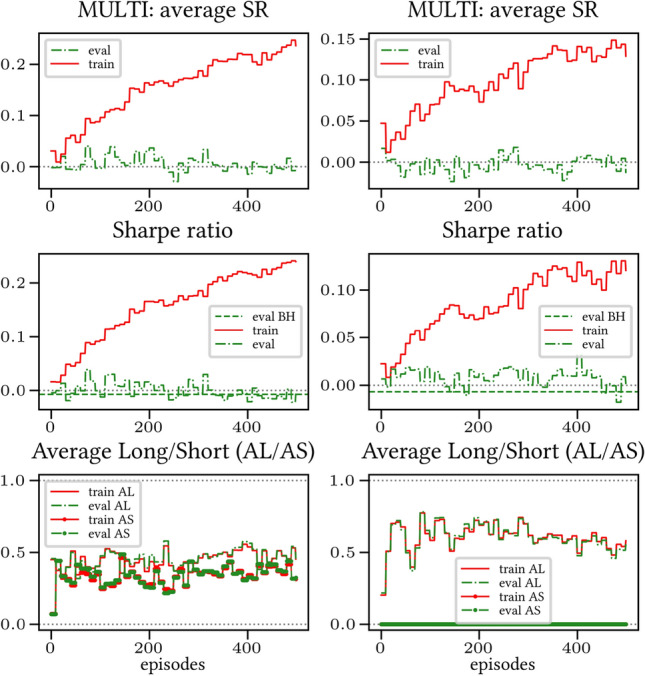
Reward Performances, SR/L &SP/BTCUSDT. Left (*Right*): Non-random (*Random*) discount factor

We have run several simulations with both fixed and randomly sampled discount factor (as suggested in [5]) on all datasets. The results are consistent across all simulations: therefore, we only show the results for the BTCUSD dataset in Figs. 21 and 22 (Multi-Reward case only, for reward SR in both the LP and L &SP cases), as they are representative of all the remaining simulations.

We were able to notice the following general trends:

- *Generalization*. A graphical comparison of performance indicators suggests that the algorithm generalizes with respect to the value of the random discount factor.
- *Training saturation levels*. There is a visible difference of saturation levels, with the random discount factor version saturating at a consistently lower level than its non-random discount factor counterpart. It is plausible that the discount factor generalization serves the purpose of a neural network regularizer. The difference is more pronounced in the L &SP case, i.e., when the agent is allowed to open Short positions.
- *Evaluation set saturation levels and average positions*. No significant differences are noticeable between the two cases.

Taking everything into account, our simulations point in the direction of validating the discount factor generalization assumption provided in [5]. Nevertheless, more extensive testing is necessary in order to fully confirm this.

### Consistent indications for predictability

Although a full statistical justification of the obtained results is beyond the scope of this paper, we nonetheless have achieved indications that validate the effectiveness and robustness of the Multi-Reward approach. We further detail this statement.

#### Consistent performance with respect to the Buy-and-Hold strategy

In the majority of simulations, the Multi-Reward is capable of improving over the Buy-and-Hold strategy benchmark (in terms of Sharpe Ratio), on the evaluation and, more importantly, on the test set (Fig. 3). Our validation has primarily relied on a distributional analysis across several experiments with independent initialization (Figs. 5, 6, 4,  19 and 3).

#### Comparing performance on training, evaluation, test sets

A commonly used model selection strategy is to pick the best performing model on the evaluation set. In Figs. 9, 10 and 11, the performance of such best performing model is shown (for training, evaluation, test) as it progresses through the episodes. We observe that the performance on the evaluation set (in terms of the Sharpe Ratio) is consistently good. In particular, the performance of the Multi-Reward model is at least as good as that of the Single-Reward model, while also being much more stable.^10^ Furthermore, the profits on the test sets are higher and more consistent in the Multi-Reward case (especially in the case of NIFTY50, see Fig. 11). The results in Figs. 9, 10 and 11 also show that the performance on the test set is loosely correlated with the one on the evaluation set. This is likely due to the noisiness of the learning process, and a neat difference between evaluation and test set. In any case, the performance on the evaluation is a reliable indication for the improved stability, as it depends on the stability of the learning process.

## Discussion and future outlook

Firstly, we have validated the generalization properties of a Multi-Reward, Reinforcement Learning method with Hindsight Experience Replay (in the declination given by [5]) by running experiments on several important, single-asset datasets (AAPL, SPY, ETHUSDT, XRPUSDT, BTCUSD, NIFTY50).

Secondly, even though a full statistical analysis would require further work, we have provided a number of consistent statistical indicators confirming the improved stability of our Multi-Reward method over its Single-Reward counterpart: distribution of performance over independent runs, convergence of prediction indicators, and profits for best performing models.

Thirdly, we verified that the Multi-Reward has a clear edge over its Single-Reward counterparts in the case of sparse, heavily position-dependent reward mechanisms.

Finally, we have partially validated the generalization property regarding the discount factor (as suggested by [5]), even though more work is required to consolidate the claim.

### Limits of the RL setting

The obtained results are, in nearly all occasions, subject to noise: more specifically, relevant performance indicators occasionally oscillate around—rather than approach—their limiting value (with respect to the number of epochs). This behavior, which is visible in the cumulative reward plots (Figs. 17, 18, 21 , 22, 7 and 8), is consistent with what can be expected from a critic-only Reinforcement Learning approach. Additionally, despite the (not at all short) length of training, evaluation, and test set, the data are obviously correlated in time, thus the effectiveness of the method is reduced. The impact of the noise is mitigated by running several experiments (Figs. 5, 6, 4,  19 and 3).

### Future outlook

While we have highlighted some of the benefits that a Multi-Reward approach has over a Single-Reward approach for predictive properties of a critic-only RL paradigm for single-asset financial data, many questions remain partially answered, or even wide open.

Firstly, while the Multi-Reward approach can stabilize and improve results of some rewards using the other ones, it is not clear exactly how the rewards are influenced by each other (normalization approaches different from Equation (3) could be investigated). Secondly, it would be interesting to conduct a more thorough research into different lengths and non-uniform sampling mechanisms for the experience replay (see [53]). Thirdly, a more thorough analysis on the use of a random discount factor should be conducted. Fourthly, one might perform a sensitivity analysis on more hyperparameters. Fifthly, a more in depth analysis of the prediction power to provide statistical evidence is still needed. Finally, it would be interesting to further address the noisy convergence of the performance metrics (see Sect. 6.1).

## Data Availability

All datasets and the entire source code used in this paper are available in open-source format at the repository https://github.com/trality/fire . In particular, the instructions for reproducibility are contained in the README.md file therein.

## Footnotes

1 Instructions on how to obtain all six datasets that we consider are available on our open-source repository https://github.com/trality/fire (see README.md file therein, and related references).

2 We stress that the term *discount factor* in this paper refers to a RL time-regularization parameter (see (1)), and should not be confused with the finance-related homonym.

3 i.e., rewards that give non-null feedback infrequently over time.

4 We use the terms ‘Multi/Single-Objective’ and ‘Multi/Single-Reward’ interchangeably throughout the paper.

5 also including the multi-agent case, which we do not treat in this work.

6 Only counting the publicly available, non-proprietary literature.

7 such an analysis is extremely tough, given that it is very hard to obtain statistically consistent positive returns in volatile single-asset problems.

8 selling previously acquired assets.

9 The average of Long positions shows the time of capital exposure to the market.

10 Further work will be needed in the future to account for the exact impact of intrinsic RL noise.
